# Reaching the unreached: Performance of an enhanced peer outreach approach to identify new HIV cases among female sex workers and men who have sex with men in HIV programs in West and Central Africa

**DOI:** 10.1371/journal.pone.0213743

**Published:** 2019-04-03

**Authors:** Tiffany A. Lillie, Navindra E. Persaud, Meghan C. DiCarlo, Dismas Gashobotse, Didier R. Kamali, Magda Cheron, Lirica Nishimoto, Christopher Akolo, Hally R. Mahler, Maria C. Au, R. Cameron Wolf

**Affiliations:** 1 FHI 360/LINKAGES, Washington, DC, United States of America; 2 FHI 360/LINKAGES, Bujumbura, Burundi; 3 FHI 360/LINKAGES, Abidjan, Cote d’Ivoire; 4 FHI 360/LINKAGES, Kinshasa, Democratic Republic of Congo; 5 U.S. Agency for International Development (USAID), Washington, DC, United States of America; University of Washington, UNITED STATES

## Abstract

Finding new HIV-positive cases remains a priority to achieve the UNAIDS goals. An enhanced peer outreach approach (EPOA) was implemented to expand the delivery of HIV services to female sex workers (FSWs) and men who have sex with men (MSM) in three countries in West and Central Africa. The aim of EPOA is to identify new HIV-positive cases. EPOA was implemented in Burundi among FSWs, and in Cote d’Ivoire and Democratic Republic of the Congo (DRC) among both FSWs and MSM. Implementation ranged from five to nine weeks and was nested within a three-month reporting period. Standard outreach was suspended for the duration of EPOA implementation but was resumed thereafter. Summary service statistics were used to compare HIV seropositivity during standard outreach and EPOA. Trends were analyzed during the quarter in which EPOA was implemented, and these were compared with the two preceding quarters. Differences in proportions of HIV seropositivity were tested using Pearson’s chi-square test; p-values of less than 0.05 were considered statistically significant. Overall, EPOA resulted in a higher proportion of new HIV-positive cases being found, both within and between quarters. In Burundi, HIV seropositivity among FSWs was significantly higher during EPOA than during standard outreach (10.8% vs. 4.1%, p<0.001). In Cote d’Ivoire, HIV seropositivity was significantly higher during EPOA among both populations (FSWs: 5.6% vs. 1.81%, p<0.01; MSM: 15.4% vs. 5.9%; p<0.01). In DRC, HIV seropositivity was significantly higher during EPOA among MSM (6.9% vs. 1.6%; p<0.001), but not among FSWs (5.2% vs. 4.3%; p = 0.08). Trends in HIV seropositivity during routine outreach for both populations were constant during three successive quarters but increased with the introduction of EPOA. EPOA is a public health approach with great potential for reaching new populations and ensuring that they are aware of their HIV status.

## Introduction

Achieving ambitious global 95-95-95 targets for HIV testing, treatment, and viral suppression requires that HIV programs find new ways to identify HIV-positive individuals and initiate them on treatment [1]. Many programs for key populations (KPs) struggle to engage people not already reached through existing program services. Existing peer outreach and community-based programs may maintain a cycle of reaching the same individuals repeatedly while not reaching others who are less visible or outside known networks.

A growing body of studies has shown that using peer mobilizers (PMs) to engage their social networks to increase the uptake of HIV testing can be effective and efficient in diagnosing new HIV infections [2–9]. Selection criteria for PMs include the strength and scope of the person’s network, geographic location, age, HIV serostatus, and adherence to antiretroviral therapy (ART) [2–4,8,10–14]. Studies have also attributed improved recruitment rates to the use of incentives [11,14]. Programs have used coupons to link successful referrals back to the recruiter, with the most common method being the use of a unique code or number [2,4,6,8,9,11,12]. In most studies, at least three coupons were given to each outreach worker, and then three more were given to each subsequent round (or wave) of PMs [2–4,7], sometimes referred to as “seeds.” Most published studies that report on the use of peer network approaches to increase testing have been conducted predominately among men who have sex with men (MSM) [2,4–8].

Linkages across the Continuum of HIV Services for Key Populations Affected by HIV (LINKAGES), a global project funded by the U.S. President’s Emergency Plan for AIDS Relief (PEPFAR) and the U.S. Agency for International Development (USAID), developed an enhanced peer outreach approach (EPOA) to expand the delivery of HIV prevention, testing, and treatment services to KPs including female sex workers (FSWs), MSM, and transgender people who have not previously engaged with HIV programs [15,16]. EPOA is based on the concept that by engaging with peers who have not formally been part of the community-based HIV program, they may reach unidentified, harder-to-reach, high-risk KP members, resulting in higher rates of seropositivity. EPOA incorporates performance-based incentives and works through social networks to improve HIV case-finding. EPOA is an additional component to an already established peer outreach program that provides a standardized prevention package (e.g., condoms/lubricants, screening for sexually transmitted infections, and psychosocial and risk-reduction counselling) for KPs.

EPOA was implemented in Burundi, Cote d’Ivoire, and Democratic Republic of the Congo (DRC) through the LINKAGES project to accelerate epidemic control in geographic areas of highest need. In West and Central Africa, as in many other regions, KPs bear a disproportionate burden of HIV infection [17–22]. UNAIDS estimates that at least 24% of new infections are among KPs in the region [22]. Moreover, West and Central Africa are behind the progress made by most regions in the world, including other parts of Africa, with only 48% of people living with HIV knowing their status [22]. Timely HIV diagnosis remains a vital step for accessing HIV treatment; however, due to the highly stigmatized and criminalized status of KPs in the region, members of these populations are often reluctant to seek and access HIV services [23–25].

Here we describe EPOA implementation in Burundi, Cote d’Ivoire, and DRC, and compare proportions of HIV seropositivity during EPOA with those during standard service delivery targeting FSWs and MSM. The aim of EPOA was to identify new HIV-positive cases among KPs so that newly diagnosed individuals could be linked to life-saving treatment for epidemic control. We found that EPOA did result in a higher proportion of new HIV-positive cases.

## Methods

In Burundi, Cote d’Ivoire, and DRC, EPOA was implemented by existing community-based organizations (CBOs) with established outreach programs that delivered services to KPs in a variety of hot spots (i.e., geographic areas where KP individuals are present and where high HIV risk behaviors sometimes take place), such as karaoke bars, short-term guest houses, massage parlors, and truck stops. The existing CBO outreach services identified, hired, trained, and paid outreach workers. The outreach workers hired by CBOs were recruited from identified hot spots, and selection characteristics included willingness to work on an HIV project, good communication and leadership skills, ability to motivate peers to seek health services, and having a medium to large social network.

**Fig 1:**
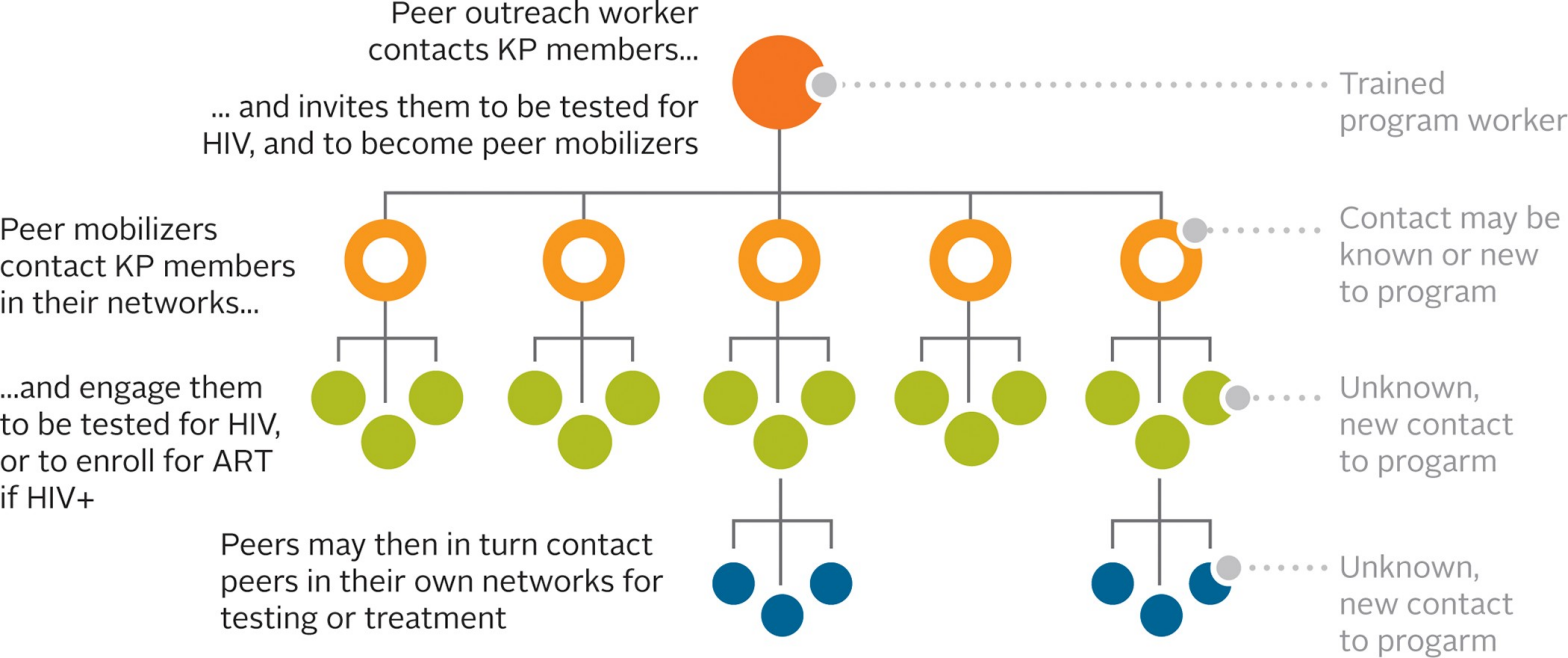
EPOA referral chain.

Through EPOA, outreach workers were trained to give coupons to PMs who were selected from within the KP community based on factors such as their network size, communication skills, risk behaviors, age, location, and knowledge of peers who engage in high-risk behaviors or had never accessed HIV services. The HIV serostatus of the outreach workers and PMs were not recorded in the study. We hypothesized that KP members reached from a PM’s social network would result in higher levels of new HIV diagnoses compared to standard outreach and testing. PMs were recruited for short-term incentivized support (i.e., unsalaried) to reach their network contacts for testing, whereas standard outreach workers were longer-term trained staff of the HIV program. PMs were not formally trained but did receive an orientation on the HIV project and EPOA process, how to select potential participants (e.g., those in their social/sexual network who would benefit from HIV services) and distribute coupons, and how they could receive an incentive. Then, PMs were incentivized if their contacts successfully accessed services and met the eligibility criteria (regardless of HIV status). The referral chain from outreach workers to PM is illustrated in Fig 1.

The EPOA design and implementation was dependent on each country’s National AIDS Program guidelines, algorithms, commodities, and established ART facilities, which resulted in country-specific variation. However, each country used the same standard operating procedures during the preparatory phase. Outreach workers were trained for three days on how EPOA is implemented, their role within the model, the procedures they would follow, and the basic skills needed to carry out their assigned tasks [16]. They were also oriented on EPOA’s goals for implementation and provided with tools for both coupon tracking and monitoring and evaluation [16]. EPOA took less than one month for the preparatory phase and then moved to the implementation phase (Table 1).

**Table 1:** EPOA implementation in Burundi, Cote d’Ivoire, and DRC, 2017.

Program components		Burundi	Cote d’Ivoire	DRC
**Implementation period**	**EPOA**	May 1–June 30, 2017	June 1–30, 2017	July 31–September 8, 2017
	**Standard**	July 1–31, 2017	April 1–May 31, 2017	September 9–30, 2017
**Implementation time frame: Coupon distribution, standard services, end of data collection**		9 weeks (May 1–June 30, 2017) out of the quarter (April 1–June 30, 2017)	5 weeks (June 1–30, 2017) out of the quarter (April 1–June 30, 2017)	6 weeks (July 31–September 8, 2017) out of the quarter (July 1– September 30, 2017)
**Geography**		• FSWs: 2 of 5 provinces (Bujumbura Mairie, Bujumbura Rural) • Mix of urban and peri-urban sites	• FSWs: 14 of 26 communes (4 communes in Abidjan, Anyama, Oume, Daloa, Issia, Bouafle, Yamoussoukro; 3 communes in Bouake, Bondoukou) • MSM: 8 of 26 communes (3 communes in Abidjan, Anyama, Agboville; 3 communes in Bouake) • Mix of urban, peri-urban, and rural sites	• FSWs and MSM: 3 of 3 provinces (Haut Katanga, Lualaba, Kinshasa) • Mix of urban, peri-urban, and rural sites
**# Community-Based Organizations (CBOs)/ Outreach Worker/Peer Mobilizer (PM)**		2 CBOs 75 outreach workers 345 PMs	3 CBOs 89 outreach workers 356 PMs	5 CBOs 201 outreach workers 148 PMs
**# of coupons distributed to each PM**		4 coupons	4 coupons	5 coupons
**Incentive structure**		• PMs, 2,000 Burundi Frac (BIF) (about US$1) per newly recruited KP individual and eligible KP individual who received HIV testing and counselling	• Outreach workers, 1000 West African Frac (CFA) (about US$2) per newly recruited KP individual and eligible KP individual who received HIV testing and counseling • Outreach workers, 500 CFA (about US$1) for each KP individual who initiated treatment • PMs, 1,500 CFA (about US$3) per newly recruited KP individual	• Outreach workers /PM, 3,000 Congolese francs (about US$2) per newly recruited KP individual and eligible KP individual who received HIV testing and counselling
**HIV testing and counselling site**		CBO facility clinics	Outreach workers at hot spots	Counselors and laboratory staff in hot spots and CBO facility clinics
**HIV test used for screening and confirmation**		Determine for screening/Dipstick for confirmation	Determine for screening/Stat-pack for confirmation	Determine for screening/Unigold for confirmation
**Antiretroviral Therapy**		Referred to CBO-led health facility	Referred to KP-friendly public health facility or a CBO-led clinic	Referred to donor-supported health facility

### Community engagement and planning

LINKAGES staff regularly consulted CBO staff and outreach workers to assess their interest in implementing EPOA and to ask for their guidance regarding service delivery approaches and appropriate incentives. An implementation plan, with an associated budget, was developed with community input to ensure consistency across in-country partners and sites.

### Site selection

Within each country, the larger geographic focus of the project was determined by the PEPFAR strategic planning processes, based on national and subnational epidemiological data and stakeholder consultation. EPOA site selection and time frames were country-specific and based on project-specific mapping and size estimation, routine program monitoring data on testing, in-country consultations, staff time, and budget.

In Burundi, EPOA was implemented with FSWs during nine weeks of the 12-week reporting period in all the sites in two (of five) provinces—Bujumbura Mairie and Bujumbura Rural (Table 1). Standard outreach was stopped in these two provinces while EPOA was implemented but resumed once EPOA concluded. Standard services were provided in the other three provinces throughout the reporting period.

Cote d’Ivoire implemented EPOA with both FSWs and MSM and focused on sites with larger KP numbers and lower testing uptake. EPOA was implemented with FSWs in 14 of the 26 communes and with MSM in eight of the 26 communes for five weeks of the 12-week reporting period (Table 1). Standard services were stopped in the selected communes for the duration of EPOA implementation but were resumed thereafter. Standard services were provided at the other communes (i.e., 12 FSW communes and 18 MSM communes) where EPOA was not implemented.

DRC implemented EPOA with FSWs and MSM in all three of the program’s provinces (i.e., Haut Katanga, Lualaba, Kinshasa) for six weeks of the 12-week reporting period (Table 1). Standard services were stopped during the six weeks in which EPOA was implemented but were resumed thereafter.

### Recruitment process and incentive

Outreach workers provided PMs with coupons to distribute to peers. Each coupon had a unique code that linked the coupon to both an outreach worker and a PM, both of whom would receive an incentive for a successful referral. Successful referrals were defined as those in which an eligible KP individual presented for services and agreed to an HIV test. (Table 1).

The coupon codes were used to track which PM networks were successful in tapping into higher-risk groups. Outreach workers provided additional coupons for more successful PMs, until they saturated their networks. Other peers within the network were also encouraged to become PMs to increase recruitment numbers. Following local and international norms, confidentiality of testing and testing results was maintained by upholding the following conditions: everyone who requested an HIV test received pre- and post-test counselling from trained counsellors; oral informed consent was obtained before the test was conducted and results were provided to the clients; then, only individuals who had not been tested in the past three months were offered an HIV test.

### Operational definitions

For the analysis, an FSW was defined as a woman who received the majority of her income in the past 12 months from goods or money in exchange for sex. A man who has sex with men was defined as a man who reported having anal sex with another man in the past 12 months. A KP individual who was “newly recruited” was an individual who met one of the above definitions but had never engaged with an HIV program. A “new HIV diagnosis” was a diagnosis in an individual who did not already know that he or she was HIV positive. Eligibility for EPOA was based on being identified as a member of a KP, not having previously engaged with an HIV program, and not having been tested for HIV in the past three months. A KP individual could also be eligible if he or she engaged with the program but had not tested for HIV in the past three months.

### Quality measures

Quality measures were implemented throughout the campaign to ensure that those included in EPOA were KP individuals, had not tested for HIV in the past three months, and had not previously been diagnosed with HIV. Standardized screening forms were used in all three countries to ensure that these criteria were met before individuals were enrolled in EPOA. Individuals who did not meet the criteria for participating in EPOA, including being HIV positive, were linked to other HIV services based on need.

### Performance monitoring

At service delivery points operated by implementing partners, routine data were collected using the standard forms for HIV prevention, testing, and treatment. During EPOA, the enrollment form was expanded to include additional information such as the coupon serial number, behavioral questions to determine KP status, whether the individual had already registered with an HIV prevention program, and if he or she had tested for HIV in the past three months. At the end of each week, partners submitted data summaries to a central program office showing the number of KP individuals who were newly recruited, tested for HIV, newly diagnosed with HIV, and initiated on ART. All data were validated on a regular basis following the established processes for data quality assurance [26].

### Data synthesis and analysis

Summary service statistics were extracted from the project reports for each country during the implementation quarter and for the two quarters preceding EPOA implementation. Data were collected on the number of individuals tested for HIV and the number of new HIV cases detected during the period of interest. In the first step of the analysis, HIV seropositivity among those recruited through EPOA was compared with those receiving standard outreach during the quarter in which EPOA was also implemented. As a second step, trends in HIV seropositivity for standard outreach during three quarters (i.e., the EPOA quarter and the two preceding quarters) were compared with trends achieved during EPOA. Differences in proportions of HIV seropositivity were calculated using the Pearson’s chi-square test; p-values of less than 0.05 were considered statistically significant.

### Ethical issues

The authors had no access to original patient records, names, or any other individual-level information. A waiver was granted by FHI 360’s institutional review board, as re-analysis of summary statistics derived from standard service delivery is not considered human subjects research.

## Results

In Burundi, 2,451 coupons were distributed to FSWs, and 929 FSWs were newly recruited and tested for HIV through EPOA. Of those tested, 100 (10.8%) were newly diagnosed with HIV. In standard outreach within the same quarter, 5,164 FSWs were tested, and 211 (4.1%) were newly diagnosed with HIV. HIV seropositivity among FSWs was significantly higher through EPOA than through standard outreach within the quarter (10.8% vs. 4.1%, p<0.001) (Table 2).

**Table 2:** HIV seropositivity in EPOA vs. standard peer outreach for FSWs and MSM in Burundi, Cote d’Ivoire, and DRC, 2017.

		Percentage (number) testing positive during a three-month period in 2017		
*Country*	*Population*	EPOA	Standard peer outreach	p-value
Burundi	FSWs	10.8% (100/929)	4.1% (211/5164)	<0.001
Cote d’Ivoire	FSWs	5.6% (194/3,476)	1.8% (106/5,840)	<0.01
	MSM	15.4% (110/714)	5.9% (93/1,569)	<0.01
DRC	FSWs	5.2% (121/2,334)	4.3% (183/4,321)	0.08
	MSM	6.9% (19/277)	1.6% (16/1,003)	<0.001

In Cote d’Ivoire, 18,796 coupons were distributed, and 3,476 FSWs and 714 MSM were newly recruited and tested for HIV through EPOA. Of those tested, 194 FSWs (5.6%) and 110 MSM (15.4%) were newly diagnosed with HIV. With standard peer outreach in the same quarter, 5,840 FSWs and 1,569 MSM were recruited and tested for HIV. Of those, 106 FSWs (1.8%) and 93 MSM (5.9%) were newly diagnosed with HIV. HIV seropositivity was significantly higher among both FSWs and MSM during EPOA than among both populations in standard outreach within the quarter (FSWs: 5.6% vs. 1.81%, p<0.01; MSM: 15.4% vs. 5.9%%, p<0.01) (Table 2).

In DRC, 2,694 coupons were distributed during EPOA, and 2,334 FSWs and 277 MSM who received coupons returned and were tested for HIV. Of those tested, 121 FSWs (5.2%) and 19 MSM (6.9%) were newly diagnosed with HIV. With standard outreach in the same quarter, 4,321 FSWs and 1,003 MSM were tested for HIV, and 183 FSWs (4.3%) and 16 MSM (1.6%) were newly diagnosed with HIV. Among MSM, HIV seropositivity was significantly higher among those recruited through EPOA than among those who received standard outreach within the quarter (6.9% vs. 1.6%, p<0.001). However, there was no statistical difference in HIV seropositivity among FSWs (5.2% vs. 4.3%; p = 0.08) (Table 2).

**Fig 2:**
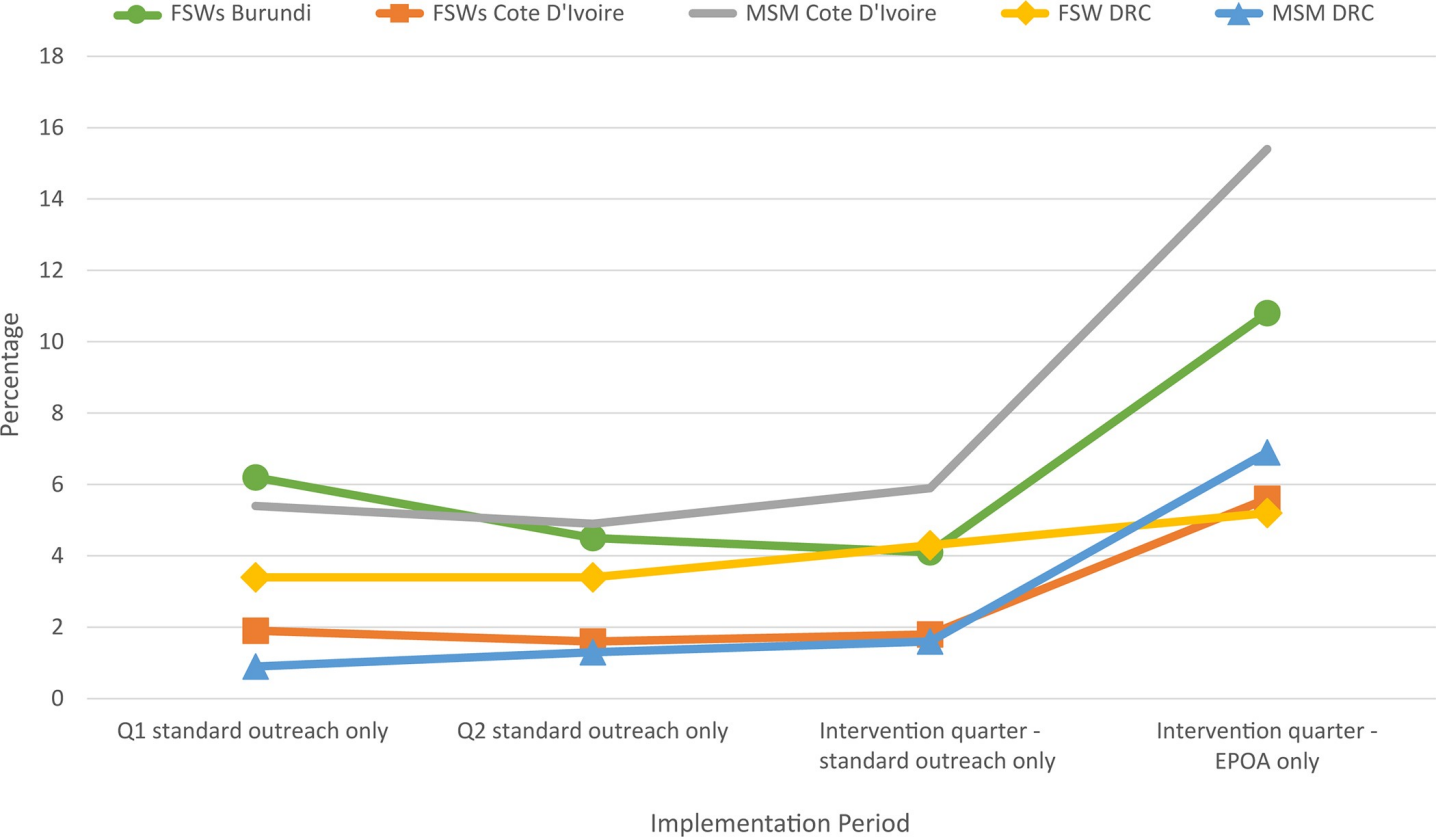
Trends in HIV seropositivity with standard outreach compared with the EPOA for FSWs and MSM in Burundi, Cote d’Ivoire, and DRC, 2017.

In all three countries, HIV seropositivity from standard outreach during successive quarters was either stable or trended downward among both FSWs and MSM. During EPOA, HIV seropositivity was higher for all populations in all three countries (Fig 2). In Burundi, HIV seropositivity among FSWs during standard outreach was 6.2% in quarter 1 (October–December 2016), 4.5% in quarter 2 (January–March 2017) and 4.1% during the intervention quarter (April–June 2017), compared with 10.8% among FSWs who participated in EPOA. In Cote d’Ivoire, HIV seropositivity among FSWs recruited using standard outreach during the three quarters was 1.9%, 1.6%, and 1.8%, compared with 5.6% among FSWs during EPOA. For MSM in Cote d’Ivoire, HIV seropositivity using standard outreach during the three quarters was 5.4%, 4.9%, and 5.9%, compared with 15.4% during EPOA. Similar trends were found in DRC, as HIV seropositivity among MSM recruited using standard outreach during the three quarters was 0.9%, 1.3%, and 1.6%, compared with 6.9% among MSM during EPOA. Finally, in DRC, HIV seropositivity among FSWs recruited using standard outreach during the three quarters was 3.4%, 3.4%, and 4.3%, compared with 5.2% among FSWs during EPOA, although results were not statistically significant.

## Discussion

These results demonstrate that during a time-bound campaign, EPOA may be more successful than standard outreach for recruiting and testing KP individuals who have a greater likelihood of HIV seropositivity. Prior to EPOA, HIV seropositivity in all three country programs remained steady. The introduction of EPOA led to increased detection of new HIV-positive KP individuals who would not have been engaged otherwise.

Additional analyses comparing EPOA results with standard outreach during the preceding two quarters were performed to address both possible temporal effects and any possible contamination. The statistical significance of the higher HIV seropositivity rate during EPOA supports EPOA as a successful way to reach those who are at higher risk and less likely to access HIV services.

The results from Burundi and Cote d’Ivoire also suggest that EPOA can be successful within the FSW population. Even though they did not reach the threshold for statistical significance, the results in DRC were similar. The majority of published studies that report on the use of enhanced peer network approaches to increase testing have been conducted among MSM [2,4–8]. Those studies that do discuss social network approaches with FSWs note challenges in recruitment due to the limited number of individuals in networks [9,27]. One explanation for the lack of statistical significance among FSWs in DRC was that outreach and testing were near saturation, which resulted in a limited number of new network members to be tested and diagnosed. Even though statistical significance was not achieved, the public health objective of identifying newly diagnosed individuals (i.e., measured by percentage of HIV case-finding) did increase from non-EPOA (standard outreach) to EPOA implementation periods.

The peer network model has also been explored within the population of people who inject drugs [13,14,28]. There is little information in the literature about using the social network model with transgender individuals in Africa; however, in the future, EPOA implementation for both people who inject drugs and transgender individuals can be explored further. While not specifically addressed in our analysis, future EPOA implementation should explore selecting KP individuals who are newly diagnosed HIV positive to act as PMs, because early results of this practice are promising in the peer-reviewed literature [29]. In addition, expanding EPOA implementation strategies for FSWs to distribute coupons to their sexual network could lead to increases in their stable (i.e., regular) and casual partners accessing HIV testing.

The greatest strengths of EPOA are that it helps penetrate previously unreached networks and delivers HIV services that can contribute toward epidemic control. EPOA also encourages outreach workers, KP individuals, and CBOs to lead the design, implementation, and continual improvement of programs. Observed challenges include managing incentives, increasing staff capacity to track and analyze the data for continual improvement in implementation, guaranteeing adequate supply of test kits, and ensuring quality control of peer implementation and data entry.

The other two vital factors that will influence the future success of EPOA are the overall coverage and saturation levels of current outreach programs and the achievement levels of EPOA in reaching KP members not engaged in the current program. If there is already high coverage and saturation within an existing program in an area, such as among FSWs in DRC, then EPOA may be less successful, since there are no new networks to engage. In the future, EPOA could be integrated and scaled with other new strategies such as pre-exposure prophylaxis for high-risk HIV-negative individuals, HIV self-testing, and index case testing among partners of KP individuals living with HIV.

### Limitations

Our analysis had several limitations. Results were based on aggregated service statistics, which, though economical, limited our analysis to only a few key indicators and did not allow the study to analyze individual-level characteristics. A more rigorous analysis of individual-level data will be needed to further determine the effectiveness of EPOA in reaching KP members with different demographic and risk characteristics, as well as the similarities and/or differences between PMs and recruited peers. Another limitation in the study is possible sampling bias in that PMs recruited peers similar to them; however, given the lack of individualized data, future analyses will have to be conducted to determine this connection. Other limitations included that standard service statistics have known challenges in data quality, and these were likely experienced within both EPOA and standard outreach data. All sites were subjected to routine data quality assurance measures developed by the program, including validation of all data reported during the performance period. Another limitation included the potential self-reporting bias of behavior when screening for eligibility to participate in the EPOA activity. Lastly, site selection was country-specific and based on in-country programmatic data, staff time, and budget; therefore, it was not random, and selection bias could have been introduced.

## Conclusion

Reaching new HIV-positive individuals is a critical component of KP programs as they strive to achieve and contribute to epidemic control. The results presented here demonstrate EPOA’s public health potential in settings such as West and Central Africa. EPOA is a promising example of how a peer-to-peer, network-based, time-bound intervention can penetrate hidden and untapped KP members and connect them to HIV services.

## Supporting information

S1 Fig Data file. (XLSX)
